# Adherence to anti-asthma medications among adult asthmatic patients in Eastern Ethiopia: A multi-center cross-sectional study

**DOI:** 10.1371/journal.pone.0277796

**Published:** 2022-12-08

**Authors:** Helina Heluf, Nega Assefa, Yadeta Dessie, Dawit Tamiru, Abel Tibebu Goshu, Gelana Fekadu

**Affiliations:** 1 School of Nursing and Midwifery, College of Health and Medical Sciences, Haramaya University, Harar, Ethiopia; 2 School of Public Health, College of Health and Medical Sciences, Haramaya University, Harar, Ethiopia; Bayero University Kano, NIGERIA

## Abstract

**Introduction:**

Adherence to anti-asthmatic medications plays a vital role in enhancing an asthma patient’s quality of life and prognosis. However, in Ethiopia, the level of adherence and contributing factors were rarely studied. Therefore, this study was conducted to determine the level of adherence to anti-asthma medications and associated factors among adult asthmatic patients in Eastern Ethiopia.

**Method:**

Institutional based cross-sectional study was conducted at six governmental hospitals found in Eastern Ethiopia. A total of 320 asthma patients aged 18 years and above and using asthma medicines for at least 12 months were involved. An interviewer based structured questionnaires were used to collect the data. Bivariable and multivariable logistic regression analyses were carried out using IBM SPSS version 22 (SPSS, Chicago, IL). The Adjusted Odds Ratio (AOR) with 95% Confidence Interval (CI) was used to determine the strength of association between independent variables and outcome variable. Variables with a p-value of ≤ 0.05 were considered statistically significant.

**Result:**

Of the 320 asthma patients that participated in the study, 109(34.1%:28.8–39.1%) of them had good adherence to anti-asthmatic medications. Being a housewife (AOR = 4.265, 95%CI: 1.333, 13.653), having good knowledge about asthma (AOR = 2.921, 95%CI (1.472, 5.795), positive attitude towards asthma (AOR = 3.129, 95%CI: 1.555, 6.293), and use of oral corticosteroid drugs (AOR = 1.967, 95%CI: 1.008, 3.841) were factors positively associated with good adherence to anti-asthmatic medications. Participants on treatment for 2–3 years (AOR = .295, 95%CI: 0.099, 0.873), and those on medication for ≥ 4 years (AOR = 0.229, 95%CI: 0.079, 0.664) were 70.5% and 77.1% times less likely to adhere to anti-asthmatic medications respectively.

**Conclusion:**

The current study signified a low level of adherence to anti-asthmatic medications. Participant’s characteristics and medication related factors were significantly associated with good adherence to anti-asthmatic medications. Health education and advice during follow-up for asthma patients is crucial for better adherence.

## Introduction

Asthma is a chronic condition that inflames and narrows the airways of the lung [1]. According to the World Health Organization (WHO) report, globally, it affected 339 million people in 2018 with the number expected to exceed 400 million by 2025 if the current rising trend continues [2–4]. Asthma is an under-diagnosed and undertreated public health problem all over the world [2]. In 2016, 10 out of one million deaths were reported to be due to asthma, with a prevalence of 50% increase per decade and it affects 5%–10% of the total population in low and middle-income countries [5–8]. Poor-adherence to pharmaceutical treatment is one of the main global challenges in asthma control and treatment [9]. Adherence is the extent to which a patient’s behavior corresponds with recommendations from a health care provider [6].

Poor adherence results in increased morbidity, health care utilization, and mortality [9, 10]. Williams et al. reported a decrease in the level of adherence by 25%, resulting in a 50% increase in the rate of asthma-related hospitalization [11].

In Ethiopia, adherence to anti-asthmatic medication was reported to be 56.7% in Gondar town [12], and 49.4% in Addis Ababa City [13]. However, according to a recent systematic review and meta-analysis results, nearly 30% of asthmatic patients in Ethiopia have poor adherence to anti-asthmatic medications [14].

Poor adherence to anti-asthmatic medications is determined by factors including lack of education on the proper use of medicines, concerns about the necessity of asthma therapy [9], route of administration, the convenience of the medication administration device, monthly income, comorbidities [13, 15, 16], adverse drug reaction, and the complexity of the drug regimen [10].

Improving medication adherence in resource-constrained countries like Ethiopia is vital for the effective management of asthma. However, only a few studies elucidate the extent of adherence to anti-asthmatic medications among asthma patients. Furthermore, these studies only focused on a patient’s level of adherence to specific types of treatment regimens; inhalation medications [13, 15], inadequate sample size and conducted at a single health facility [10, 12, 17] yielding unrepresentative data. Therefore, the current study was conducted at six different hospitals with adequate sample size, and includes all types of anti-asthmatic medications to assess the level of adherence to anti-asthmatic medications and associated factors among asthmatic patients attending governmental hospitals in Eastern Ethiopia.

## Methods

### Study setting and population

A hospital-based cross-sectional study was conducted from February 21 to April 20, 2020. Study participants were recruited from six government hospitals, namely; Hiwot Fana Comprehensive Specialized Hospital, Jugol General Hospital, and Federal Police Harar General Hospital in Harar region, as well as Dilchora Referral Hospital, Sabian General Hospital, and East Command Level Referral Hospital in Dire Dawa City Administration.

The diagnosis of asthma was based on medical history, physical examination, and spirometer result (based on the Global initiative for asthma diagnosis criteria) [18]. The study population were asthmatic patients who fulfilled the inclusion criteria and were presented at the selected hospitals for follow-up during the data collection period. Inclusion criteria were asthma patients aged 18 years and above and using asthma medicine for at least 12 months. Asthmatic patients who were critically ill and those with severe mental illness, or unable to respond to the questionnaire, as well as those with physician-diagnosed active lung infections, and Chronic Obstructive Pulmonary Disease (COPD) were excluded from the study.

### Sample size and sampling procedure

The sample size was calculated using the single population proportion formula [19] by considering the confidence level of 95%, margin of error of 5%, and proportion of adherence to anti-asthma medication 33.8% [20]. Accordingly, the final sample size was 343. Participants were recruited from each facility until the allotted number was reached.

### Data collection procedures and tools

Data was collected by six nurses with bachelor’s degrees under the supervision of a nurse with a master’s degree. An interviewer-based structured questionnaire was used.

The questionnaires were first prepared in English, and were translated to the local languages (Amharic, Afaan Oromo, and Af Somali) and translated back to English, whilst ensuring that conceptual equivalence was maintained. The questionnaire included items pertaining to sociodemographic characteristics (sex, marital status, educational status, occupation, residence, and wealth index (a measure of participant’s socio-economic status), medication adherence, types of medications used, patient-related factors (having regular follow-up, history of cigarette smoking and chewing khat, attitude towards asthma, knowledge about asthma and family history of asthma), disease-related factors (presence of exacerbation in the past 12 months, comorbidity, and exacerbating factor, history of hospital admission, and duration of treatment).

The Medication Adherence Reporting scale (MARs) that includes ten questions was used to assess the participant’s level of drug adherence. The tool was used as it shows good construct, internal, and criterion validity, and was employed by previous locally constructed studies [21, 22]. It includes both generic “I use it regularly every day” and asthma-specific questions about medication use “I only use it when I feel breathless”; It also assesses both intentional “I avoid using it if I can”, and unintentional non-adherence “I forget to use it” [23]. In order to minimize the social desirability bias, the questions were framed as negative statements. The level of medication use was rated on a five-point Likert scale (1; always to 5; never). Self-reported adherence was reported as the average score of the 10 items (1–5), where higher scores indicate good levels of reported adherence. Good adherence was defined as a MARs score of 4.5 or higher [21, 24, 25].

The knowledge part of the questionnaire included 20 questions. Participant’s scores of at least 50% of each field of knowledge were considered to have good asthma knowledge, and those participants’ scores less than 50% were considered to have poor knowledge [26].

The attitude section of the questionnaire included ten questions framed as a positive statement and were scored on a five-point Likert scale ranging from strongly disagree to strongly agree. Accordingly, 1 was given for strongly disagree, 2 for disagree, 3 for neutral, 4 agree, and 5 for strongly agree. Participants with a score ≥ 30 out of 50 were judged to have a positive attitude, while those with a score < 30 were regarded to have a negative attitude [27, 28].

### Data quality control

Pretest of the questionnaire was conducted on 5% (16) of the sample size at Haramaya Hospital before the actual data collection. Relevant changes were made to the questionnaire after the pretest. Two days of training were given for research assistants and supervisor on the research objectives, data collection tools, procedures, and interview techniques. Data were checked for completeness, consistency, and double data entry done for cross-validation.

### Data processing and analysis

Data was checked for completeness and inconsistencies before entering into Epi-data version 3.1.0 and exported to IBM SPSS version 22 (SPSS, Chicago, IL) for analysis. Descriptive statistics including frequencies, proportion, mean, and Standard Deviation (SD) were computed. A wealth index using principal component analysis was carried out to determine participant’s socio-economic status. The participant’s household wealth was ranked into three quartiles; low, medium, and high. Binary logistic regression analysis was done and all explanatory variables with a p value less than 0.25 on a bivariable logistic regression analysis were entered into a multivariable logistic regression model to identify factors associated with adherence to anti-asthma medications [29, 30]. The Crude Odds Ratio (COR) and Adjusted Odds Ratio (AOR) along with 95% Confidence Interval (CI) were calculated to measure the strength of association between the independent and outcome variable (level of adherence to anti-asthmatic medications). The fitness of the model was tested by the Hosmer-Lemshow test and was considered fit because it yielded a p value of greater than 0.05 [31, 32]. Thus, a p value of less than 0.05 was considered to declare the presence of statistical significance.

### Ethical approval

This study was conducted in accordance with the Declaration of Helsinki’s ethical principles for medical research involving human subjects. Accordingly, ethical clearance was obtained from Haramaya University, College of Health and Medical Sciences, Institutional Health Research Ethics Review Committee (IHRERC) with reference number (IHRERC/014/2020). An official letter was sent to the selected hospitals for permission to recruit asthma patients. The study participants provided informed, voluntary, and written consent, and they were notified of their right to withdraw their participation in the study at any time. All personal identifiers were excluded; all information obtained was kept confidential, and used for the proposed study only.

## Results

### Socio-demographic characteristics

A total of 320 individuals participated in the study and a response rate of 93.2% was achieved. The mean age of the participants was 49.9 (±13.39) years, with the range being 18–76 years. Of the total participants, 169(52.8%) were females and 190 (59.4%) were married. One hundred twelve (35%) participants had attended higher education, 160(50%) of them were private employees, and 107 (33.4%) were in the category of high socio-economic status (Table 1).

**Table 1:** Socio-demographic characteristics of study participants.

*Variable*	*Response*	*Frequency*	*Percent*
Sex	Male	151	47.2
	Female	169	52.8
Marital status	Single	72	22.5
	Married	190	59.4
	Divorced	25	7.8
	Widowed	33	10.3
Educational status	Unable to read and write	31	9.7
	Primary education	94	29.4
	Secondary education	83	25.9
	Higher education	112	35.0
Occupation	Government employee	88	27.5
	Private employee	160	50.0
	Housewife	51	15.9
	Unemployed	21	6.6
Residence	Urban	298	93.1
	Rural	22	6.9
Wealth quartile	Low	106	33.2
	Medium	107	33.4
	High	107	33.4

### Level of medication adherence

Regarding medication adherence, only 109 (34.1%:28.8–39.1%) of the study participants had good adherence to anti-asthmatic medications. Female participants had a higher level of adherence 60(55%) than their male counterparts. Participants who were married had a higher levels of adherence 65(59.7%), followed by single 28(25.7%), widowed 11(10%), and divorced 5(4.6%). Similarly, good adherence level to anti-asthmatic medications according to the wealth index were 31(28.4%) for low, 38(34.8%) for medium, and 40(36.6%) for high.

### Types of medications

**Fig 1:**
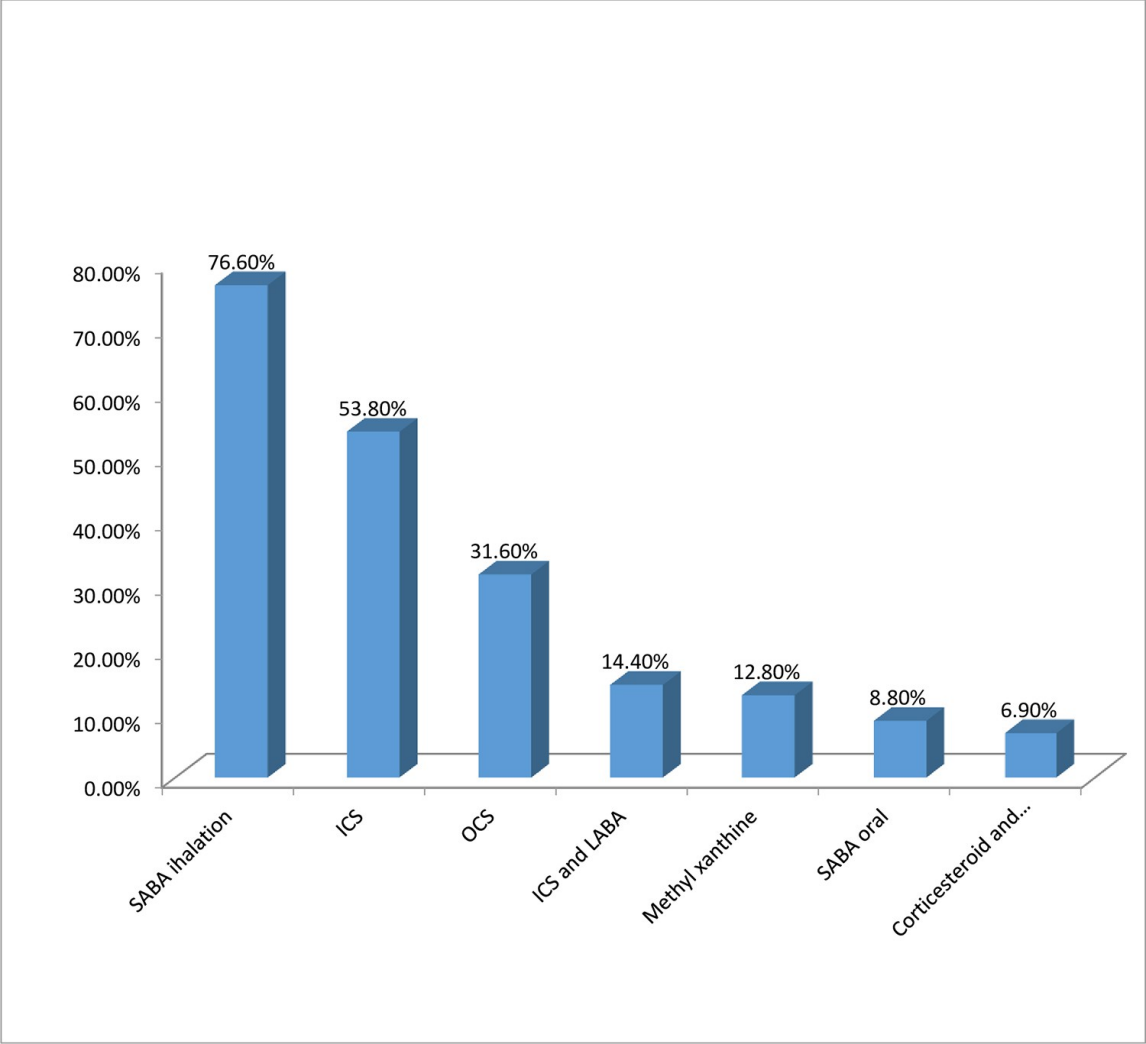
Type of medications used by study participants. Note; SABA: Short Acting Beta2 Agonist, ICS; Inhaled Corticosteroid, OCS; Oral Corticosteroid, LABA; long acting Beta2 Agonist.

The majority of participants, 245(76.6%) used inhaled-short acting beta 2 agonist, followed by inhaled corticosteroids, 172(53.8%) and oral corticosteroids, 101 (31.6%) to control asthma (Fig 1).

### Patient-related factors

Of the total study participants, 22(38.1%) attended their regular medical follow-up. The majority of the study participants, 289(90.3%) had never smoked cigarettes and 185(57.8%) of them had the habit of chewing khat. Regarding the knowledge and attitude of the participants towards asthma, 127(39.7%) had poor knowledge about asthma and 165(51.6%) had a positive attitude towards asthma (Table 2).

**Table 2:** Patient related characteristics of study participants.

*Variable*	*Response*	*Frequency*	*Percent*
Regular medical follow-up	Yes	122	38.1
	No	198	61.9
Smoking	Yes	185	57.8
	No	135	42.2
Khat chewing	Yes	185	57.8
	No	135	42.2
Knowledge about asthma	Good knowledge	199	62.0
	Poor knowledge	121	38.0
Attitude towards asthma	Positive attitude	165	51.6
	Negative attitude	155	48.4
Presence of family history of asthma	Yes	113	35.3
	No	207	64.7

### Disease related factors

Almost half of the study participants, 155(48.4%), had experienced asthma exacerbation in the past 12 months. Regarding the presence of comorbidity, 130(40.6%) had a comorbid illness and 211(90.2%) participants reported that upper respiratory tract infections precipitated their asthma attack. Only 36(11.3%) of participants reported a history of hospitalization in the previous 12 months, with 17(47.2%) of these admissions attributable to asthma (Table 3).

**Table 3:** Disease related factors among study participants.

*Variable*	*Response*	*Frequency*	*Percent*
Presence of exacerbation in the past 12 months	Yes	155	48.4
	No	165	51.6
Presence of comorbid illness	Yes	132	41.2
	No	190	58.8
Types comorbid illness	Allergic rhinitis	14	8.0
	Diabetes mellitus	75	42.7
	Hypertension	62	35.3
	Human Immune deficiency virus	16	9.0
	*Others	9	5.0
Presence of precipitating factor	Yes	234	73.1
	No	86	26.9
Types of precipitating factor	Gastro esophageal reflux disease	67	28.6
	Upper respiratory tract infection	211	90.2
	Urinary tract infection	13	5.6
Hospital admission in the past 12 months	Yes	36	11.3
	No	284	88.8
Reason for hospital admission	Asthma	17	47.2
	**Others	19	52.8
DTFA	≤1 year	26	8.1
	2–3 years	102	31.9
	≥4 years	192	60.0

*Others: hepatitis, thyroid disease, breast cancer, chronic kidney disease

** Others; Diabetic ketoacidosis, appendicitis, traumatic injury, peptic ulcer disease, benign prostate hyperplasia and anemia, DTFA: Duration of treatment for asthma.

### Triggering factors of asthma

**Fig 2:**
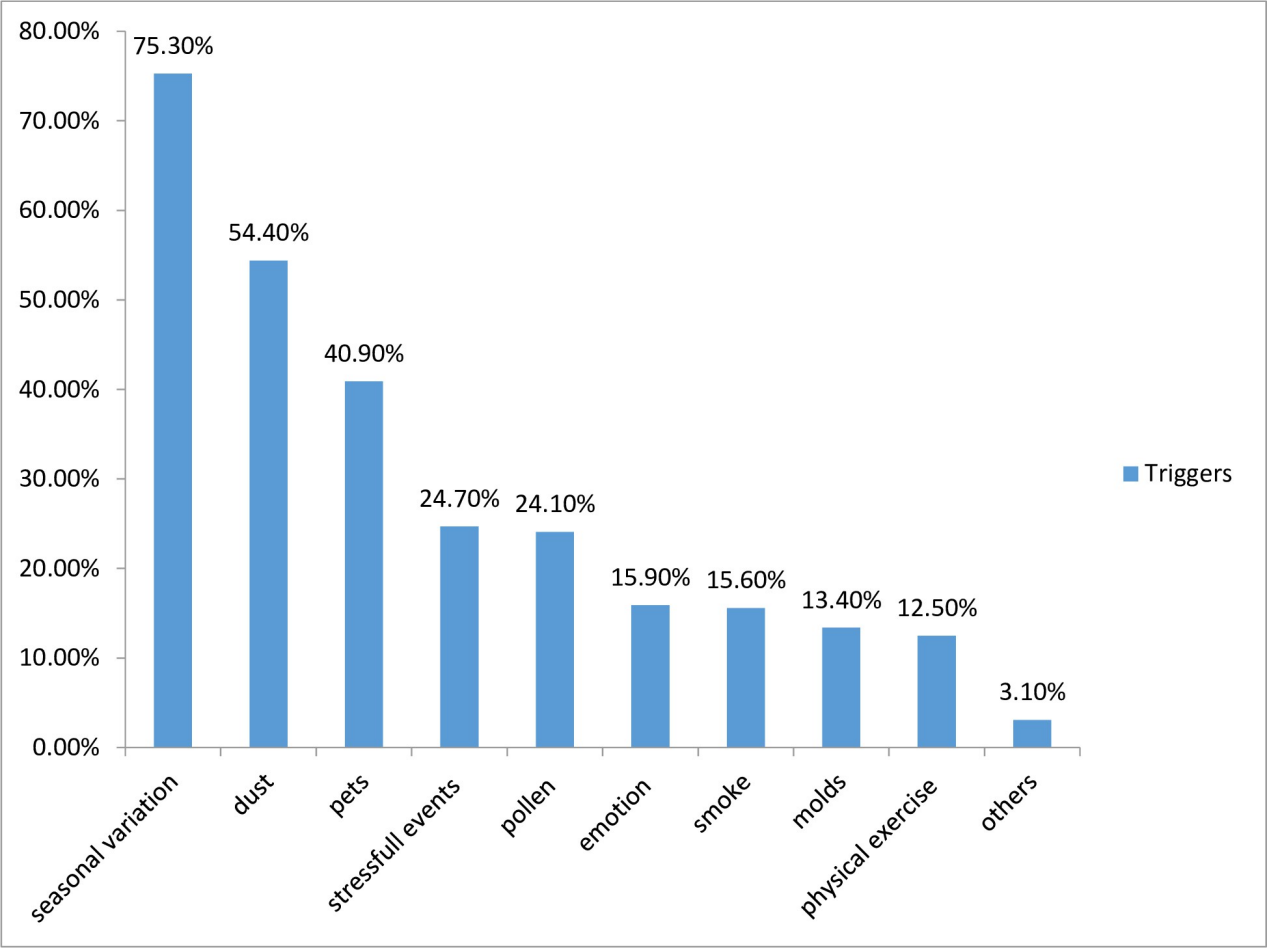
Triggering factors of asthma among study participants. Note; Others: perfume, aspirin, certain food, work related trigger (working in industry), and beta-blockers.

Majority, 241(75.3%) of the study participants reported a seasonal variation as the major triggering factor followed by dusts 174(54.4%) and pets 131(40.9%) (Fig 2).

### Factors associated with adherence to anti-asthmatic medications

Factors associated with adherence to anti-asthmatic medications were identified. Age, educational level, occupation, knowledge about asthma, attitude towards asthma, chewing khat, having regular follow-up, using short-acting beta-agonist drugs, using an oral corticosteroid, presence of comorbidities, presence of precipitating health condition, admission in the previous 12 months, and duration of asthma treatment were all associated with adherence to anti-asthmatic medications under bivariable analysis. With further analysis, occupation, knowledge about asthma, attitude towards asthma, using oral corticosteroid drugs, and the duration of asthma treatment were shown to be significantly associated with the outcome variable.

Those participants who were housewives were 4.27 times (AOR = 4.27, 95%CI: 1.33, 13.65) more likely to adhere to their anti-asthmatic medications compared to government employees. Furthermore, participants with good asthma knowledge were 2.92 times (AOR = 2.92, 95%CI: 1.47, 5.79) more likely to adhere to anti-asthmatic medications than those with poor asthma knowledge. Similarly, participants with a positive attitude towards asthma were 3.13 times (AOR = 3.13, 95%CI: 1.55, 6.29) more likely to adhere to anti-asthmatic medications than their counterparts with a negative attitude. The odds of good adherence were 1.97 times (AOR = 1.97, 95%CI: 1.00, 3.84) higher for participants on oral corticosteroids than for those on other types of medications. Long-term use of anti-asthmatic medications was associated with a lower level of adherence (Table 4).

**Table 4:** Factors associated with adherence to anti-asthmatic medications among study participants.

*Variables*	*Response*	*Adherence*		*95%CI*	
		*Poor (%)*	*Good (%)*	*COR*	*AOR*
Age in years	Mean ± SD 49.9±13.39	211(66)	109(34)	0.976(0.95,0.99)	0.99(0.96,1.01)
Educational level	Unable to read and write	26(84)	5(16)	1	1
	Primary	60(64)	34(36)	2.94(1.03,8.38)	2.07 (0.56,7.17)
	Secondary	59(71)	24(29)	2.11(1.12,3.99)	1.82 (0.47,7.023)
	College and above	66(59)	46(41)	3.62(1.29,10.13)	3.00(0.68,13.21)
Occupation	Government employee	57(65)	31(35)	1	1
	Private employee	108(81.2)	52(18.8)	0.88(0.51,1.53)	1.75(0.72,4.31)
	Housewife	29(56.8)	22(43.2)	1.39(0.68,2.82)	4.27(1.33,13.65)*
	Unemployed	17(81)	4(19)	0.433(0.13,1.39)	0.52 (0.13,2.15)
Knowledge about asthma	Poor	99(81.8)	22(18.2)	1	1
	Good	112(56)	87(44)	3.50(2.40,6.00)	2.92 (1.47,5.79)*
Chewing khat	No	79 (58.5)	56 (41.5)	1	1
	Yes	132 (71.4)	53 (28.6)	0.56(0.35,0.90)	1.05(0.57,1.93)
Having regular follow-up	Yes	74(60.6)	48(39.4)	1	1
	No	137(70)	61(30)	0.68(0.43,1.10)	0.65(0.33,1.29)
Attitude towards asthma	Negative	121(78.1)	34(21.9)	1	1
	Positive	90(54.5)	75(45.5)	2.96(1.82,4.83)	3.13 (1.55,6.29*
Use of Short acting beta 2 agonists	No	42(56)	33(44)	1	1
	Yes	169(69)	76(31)	0.57(0.34,0.97)	1.38(0.71,2.70)
Use of Oral corticosteroids	No	133(60.7)	86(39.3)	1	
	Yes	78(77.2)	23(22.8)	0.46(0.27,0.78)	1.97(1.00,3.84)*
Comorbidities	No	116(61.1)	74(38.9)	1	1
	Yes	95(73.1)	35(26.9)	0.57(0.34,0.97)	0.90(0.45,1.83)
Presence of precipitating factors	No	51(59.3)	35(40.7)	1	1
	Yes	160(68.3)	74(31.7)	0.67(0.40,1.12)	0.89(0.45,1.78)
Admission in the past 12 months	No	181(63.7)	103(36.3)	1	1
	Yes	30(83.3)	6(16.7)	0.35(0.14,0.87)	1.93(.60,6.18)
Duration of treatment for asthma	≤1 year	13(50)	13(50)	1	1
	2–3 years	72(70.5)	30(29.5)	0.42(0.17,1.00)	0.295 (0.09,0.87)*
	≥4 years	126(65.6)	66(34.4)	0.52(0.31,0.88)	0.22(.08,0.66)*

Note: CI; Confidence Interval, COR; Crude Odds Ratio, AOR; Adjusted Odds Ratio, *; Significantly associated variable

## Discussion

In this study, the percentage of good adherence to anti-asthmatic medications was 34.1% (CI: 28.8%-39.1%). This is in line with studies conducted in the United States of America and Southern Ethiopia, where the percentage of good adherence to anti-asthma medications were 33.8% and 40.8% respectively [17, 20]. However, this finding is higher than the result of a study conducted in Northern Ethiopia, where the percentage was 22.7% [12]. However, the current finding is lower than the result of studies conducted in Kenya, 51.5% [33], and in Western Ethiopia, 62.1% [10]. The difference in sample size may contribute to the discrepancy.

In our finding, participants identified as housewives were more likely to adhere to anti-asthmatic medications than their government employee counterparts. This finding is in contrast to previous results which have shown no significant association between occupation and medication adherence [12, 17, 34, 35]. The finding of the current study could be linked to the fact that housewives could be able to take their medicines on time since they mostly stay at home and visit their doctors regularly, unlike government employees, who may be busy at work. Also, Tomar et al. [36], revealed that under-educated patients were more adherent to anti-asthmatic medications prescribed by health care providers, whereas those who have a better education had a second opinion and decided to alter their treatment. The current study results indicate that participants who had good knowledge of asthma were more likely to have good adherence to anti-asthmatic medications than their counterparts. This finding is in line with previous studies [10, 33], which showed that asthmatic patients who had poor knowledge about their disease condition were more likely to be non-adherent to anti-asthmatic medications as compared with those who had good knowledge about asthma.

Patients who had a positive attitude towards asthma were more likely to adhere to the anti-asthmatic medications than their counterparts with a negative attitude towards asthma. This finding is supported by the results of a randomized control trial that shows that a positive attitude towards asthma increased the level of medication adherence [37]. In contrast, another study from Southern Ethiopia, shows no significant association between the attitude of asthmatic patients towards asthma and adherence to anti-asthmatic medication [10].

Participants on oral corticosteroid types of medications have better adherence than those on other types of drugs. In contrast, studies conducted in Cameroon and Ethiopia outlined no association between the types of drugs used and the level of adherence to anti-asthmatic medications [38, 39]. This discrepancy could be related to the difference in the categories of medications taken by participants, with the current study including seven types of medications while the previously conducted studies included only three categories of anti-asthmatic medications.

In the current study, receiving anti-asthmatic medications for a longer period of time was found to be associated with poor medication adherence. According to the study conducted in Cameroon, there was no association between the duration of asthma treatment and adherence to anti-asthmatic medications [38]. This may be related to the fatigue that occurs after taking drugs for an extended period of time [12].

### Strengths and limitations

In the current study, standardized, reliable, and validated tools were used to assess patient’s level of medication adherence. However, the cross-sectional nature of this study does not show a cause-and-effect relationship between the outcome and independent variables, and the level of medication adherence was assessed by self-report, which may have incurred recall bias. Moreover, the study was not able to illuminate the reason for poor anti-asthmatic medication adherence.

## Conclusion and recommendations

This study has demonstrated a low level of adherence to anti-asthmatic medications among participants. Also, we identified personal and medication related factors associated with good adherence to anti-asthmatic medications. Enhancing the level of medication adherence is crucial for the treatment and control of asthma. Health care providers have to counsel and teach asthmatic patients about the benefits of adherence to anti-asthmatic medications. Moreover, we recommend that future researchers to address the limitations of our study through a qualitative approach or mixed-method.

## Supporting information

S1 Data (SAV)
